# Evaluation of extra capsular lymph node involvement in patients with extra-hepatic bile duct cancer

**DOI:** 10.1186/1477-7819-10-106

**Published:** 2012-06-08

**Authors:** Takehiro Noji, Masaki Miyamoto, Kanako C Kubota, Toshiya Shinohara, Yoshiyasu Ambo, Yoshihiro Matsuno, Nobuichi Kashimura, Satoshi Hirano

**Affiliations:** 1 Gastrointestinal Surgery II, Department of Surgery, Hokkaido University Graduate School of Medicine, N15 W7, Kita-ku, Sapporo, 060-8638, Japan; 2 Department of Surgery, Teine-Keijinkai Hospital, 1-40 Maeda1-12, Teine-ku, Sapporo, 006-8555, Japan; 3 Department of Surgical Pathology, Hokkaido University Hospital, N14 W5, Kita-ku, Sapporo, 060-8648, Japan; 4 Department of Pathology, Teine-Keijinkai Hospital, 1-40 Maeda1-12, Teine-ku, Sapporo, 006-8555, Japan

**Keywords:** Extra-capsular lymph node involvement, Extra-hepatic bile duct cancer, Subgroup of lymph node metastasis

## Abstract

**Background:**

Lymph node metastasis is one of the most important prognostic factors for extra-hepatic bile duct carcinoma (ExHBDC). Extra capsular lymph node involvement (ExCLNI) is the extension of cancer cells through the nodal capsule into the perinodal fatty tissue. The prognostic impact of ExCLNI has been shown to be significant mainly in head and neck malignancies. Recently, the prognostic impacts of ExCLNI have evaluated in gastrointestinal malignancies. However no data is available regarding the incidence and prognostic significance of extra-capsular lymph node involvement (ExCLNI) in resectable ExHBDCs. The aim of the present study is first to evaluate the incidence of ExCLNI in surgically-treated ExHBDCs and second, to determine the prognostic impact of ExCLNI in patients with surgically-treated ExHBDCs.

**Methods:**

A total of 228 patients (110 cases of hilar cholangiocarcinoma and 118 cases of distal cholangiocarcinoma) with surgically-treated ExHBDCs were included in this retrospective study. ExCLNI was defined as the extension of cancer cells through the nodal capsule into the perinodal fatty tissue. The existence of ExCLNI and its prognostic value were analyzed as a subgroup of lymph node metastasis.

**Results:**

ExCLNI was detected in only 22% of patients with lymph node metastasis of surgically-treated ExHBDC. The presence of ExCLNI correlated with distal cholangiocarcinoma (*p* = 0.002). On univariate analysis for survival, perineural invasion, vascular invasion, histological grade, and lymph node metastasis were statistically significant factors. On multivariate analysis, only lymph node metastasis was identified as a significant independent prognostic factor in patients with resectable ExHBDC. Subgroups of lymph node metastasis including the presence of ExCLNI, location of lymph node metastasis, and the number of lymph node metastasis had no statistically significant impact on survival.

**Conclusion:**

ExCLNI was present in only 22% of the LNM (7% of overall patients) in patients with surgical treated ExHBDCs. And ExCLNI would have no impact on the survival of patients with surgically-treated ExHBDCs.

## Background

Extra-hepatic bile duct cancers (ExHBDCs) continue to be one of the most difficult cancers to manage in terms of staging and radical resection. Despite this fact, preoperative diagnosis, management, and operative strategy have improved. The 5-year survival rate still ranges from 22% to 40% [1-11]. Regional and para-aortic lymph nodes are frequently involved in ExHBDCs. Lymph node metastasis (LNM) is still one of the most important prognostic factors, although extended lymphadenectomy provides survival benefits in selected patients with ExHBDCs [6-8,11-13]. Recently, several investigators have shown that the subcategories of LNM such as location of the LNM, the number of LNMs, and the metastatic lymph node ratio (LNR) were significant prognostic factors [6,11,14-16].

Extra capsular lymph node involvement (ExCLNI) is the extension of cancer cells through the nodal capsule into the perinodal fatty tissue. The prognostic impact of ExCLNI has been shown to be significant mainly in head and neck malignancies. Recent studies have evaluated the prognostic value of ExCLNI in other gastrointestinal malignancies such as esophageal, colon, and gastric cancer, adenocarcinoma of the ampulla of Vater, and pancreatic carcinoma [17-22].

Several authors have stated that the presence of ExCLNI has a prognostic impact based on the following rationale:

a) ExCLNI could reflect the invasiveness and aggressiveness of the primary tumor [23,24] and

b) ExCLNI could be one of many patterns of cancer dissemination [23].

In our best knowledge, no data are available on incidence and the prognostic significance of ExCLNI in resectable ExHBDCs [23].

The aim of the present study was to first evaluate the incidence of ExCLNI in surgically treated ExHBDCs, and second, to determine the prognostic impact of ExCLNI (including other subgroups of LNM) in patients with surgically treated ExHBDCs.

## Methods

### Patients

Curative resections were performed on 139 patients with ExHBDCs at the Second Department of Surgery, Hokkaido University Hospital in Japan from December 1999 to November 2007. An additional 89 patients with ExHBDCs, who underwent resection at Teine-Keijinkai Hospital, Japan from January 1997 to November 2007, were also included. A total of 152 men and 76 women were included in this study. The median age was 70 (range 20 to 85). There were 110 cases of hilar cholangiocarcinoma and 118 cases of distal cholangiocarcinoma. Thus, a total of 228 patients with ExHBDCs were included in this report. Patients who underwent an exploratory laparotomy, or were diagnosed with gallbladder cancer, adenocarcinoma of the ampulla of Vater, or intra hepatic bile duct cancer in the postoperative period were excluded. Clinicopathologic features of patients are shown in Table 1.

**Table 1:** Clinicopathological features of 228 cases of extra hepatic cholangiocarcinoma

**Clinicopathological characteristics (N = 228)**			**Tumor location**	
			**Hilar n = 110**	**Distal n = 118**
Age(years)	>70	126	69	57
Gender	Male/Female	152/76	85/25	67/51
Surgical procedure	Major hepatectomy	90	81	9
	P.D	95	8	87
	P.D with major hepatectomy	14	8	6
	Extra hepatic bile duct resection	29	13	16
pT Factor	T1-2	91	46	45
	T3-4	137	64	73
Lymph node metastasis	Present/Absent	79/149	41/69	38/80
Vascular invasion	Present/Absent	46/182	35/75	11/107
Perineural invasion	Present/Absent	184/44	92/18	92/26
Histological grade	G1	64	27	37
	G2	128	63	65
	G3	33	19	14
	Others	2	1	2
Microscopic margin status	Positive/Negative	29/199	16/94	13/105

Clinicopathological characteristics of patients in this study. pT: 7th classification of AJCC pathological T factor P.D: Pancreaticoduodenectomy.

Tumors were classified by the tumor-node-metastasis (TNM) classification criteria according to the American Joint Committee on Cancer (AJCC), 7th edition [25]. Classifications of lymph node groups according to location of the LNM in the present study were classified according to the 5^th^ edition of Japanese General Rules for Classification of Biliary Tract Carcinoma currently used in Japan [26]. This classification system was used to define the topographic relations of lymph nodes to surrounding structure. For example, lymph node posterior to the portal vein (12p) is defined as group 1 lymph node (N1) in hilar cholangiocarcinoma, but the same lymph node was defined as group 2 lymph node (N2) in inferior cholangiocarcinoma (See reference [26]).

### Operative procedure and adjuvant therapy

The operative criteria and procedure for ExHBDCs were described in previous reports [1,27-29]. Some of the patients with T4 (AJCC), whose disease had spread to the main or bilateral branches of the portal vein, were considered unrespectable by many surgeons, but were candidates for resection in our institutes [1,29].

Patients with hilar cholangiocarcinoma were mainly treated by major hepatectomy with skeletonization of the portal vein and hepatic artery, and with nodal clearance around the head of the pancreas. All hepatectomy included a caudate lobectomy. Distal cholangiocarcinoma were mainly treated by pancreaticoduodenectomy (SSPPD) with en-bloc resection of the primary tumor and regional lymph nodes of the hepatoduodenal ligament. Skeletonization of the portal vein and the hepatic artery was included in this procedure as well. An extra hepatic bile duct resection was performed for selected patients with liver dysfunction or other complications. No definitive chemotherapy or radiation was added after surgery.

Multiple resections were performed including 90 cases of major hepatectomy (31 cases of left and 52 cases of right hepatectomy, 2 cases of left and 5 cases of right trisegmentectomy), 95 cases of pancreaticoduodenectomy (including 4 cases of caudate lobe resection), 13 cases of pancreaticoduodenectomy with major hepatectomy, and 29 cases of extra-hepatic duct resection (Table 1).

### Pathological diagnosis and analysis

Our pathological diagnosis protocol and analysis for ExHBDCs were based on prior studies [12,13,30]. Lymph nodes were counted and measured with a slide gauge. All surgically dissected lymph nodes were examined pathologically for metastatic foci. For each patient with LNM, all available hematoxylin and eosin stain slides of lymph nodes were re-evaluated on a double-headed microscope by three investigators (MM, TN, and KK) until a consensus was reached. All investigators were blinded to survival data.

**Figure 1:**
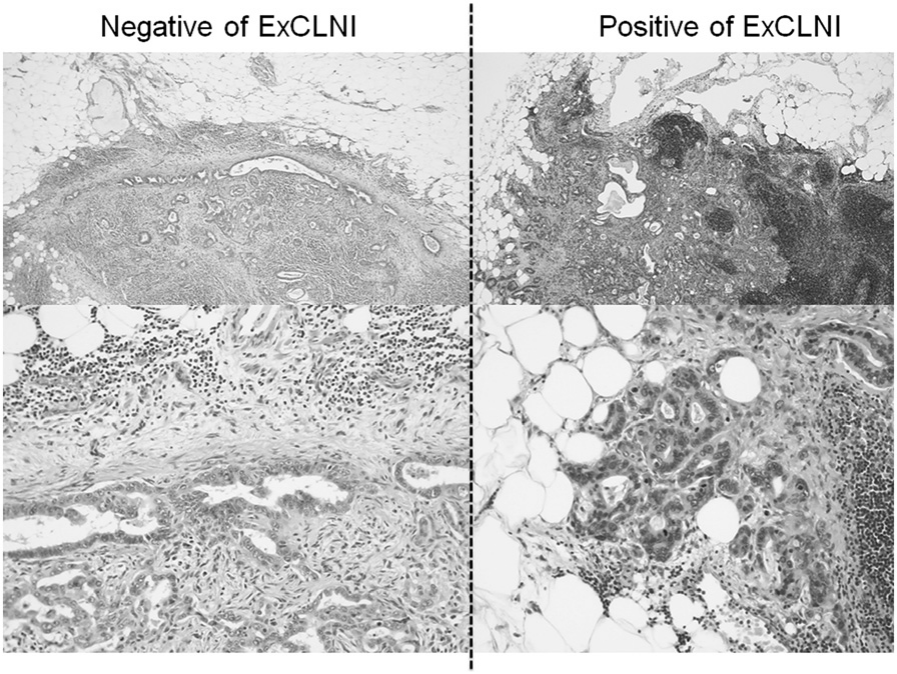
**A lymph node with intra-capsular tumor involvement embedded in perinodal fat tissue is shown on the left side (upper: low power field and lower: high power field).** An adenocarcinoma extending through the capsule into the perinodal fat tissue is shown on the right (upper: low power field and lower: high power field).

The following data were collected for the resected specimens: tumor differentiation, perineural invasion, lymph node status, portal vein and hepatic artery invasion (vascular invasion), and resection margin status. ExCLNI was defined as the extension of cancer cells through the nodal capsule into the perinodal fatty tissue (Figure 1). We used lymphatic endothelial marker (D2-40) and Elastica-Masson stain, when we found a difficulty to distinguish lymphatic invasion and venous invasion from ExCLNI. Cases in which the cancer extended into afferent and efferent structures such as lymphatic or blood vessels were strictly excluded.

### Statistical analysis

Statistical calculations were performed using Dr SPSS-II (SPSS, Chicago, Illinois, USA). The Chi-square, Fisher’s exact, and Mann–Whitney U tests were used as appropriate. Cumulative survival after surgery was calculated using the Kaplan-Meier method. The log-rank test was used to compare cumulative survival. Cox proportional regression hazard model was used for multivariate analysis. Values of *p* <0.05 were considered statistically significant.

## Results

### Clinicopathological results

Median total lymph nodes counts resected were 14 in our presented study. There were 79 patients with LNM. There were 41 cases of hilar cholangiocarcinoma and 38 cases of distal cholangiocarcinoma with LNM, respectively.

Median number of LNM were 2 (range 1–8) in hilar cholangiocarcinoma, and 2 (range 1–6) in distal cholangiocarcinoma, respectively. Among 79 patients, there were 49 cases of N1, 24 case of N2, 5 cases of N3 patients. (There were 23 cases of N1, 13 cases of N2, and three cases N3 patients in hilar cholangiocarcinoma patients. There were 26 cases of N1, 12 cases of N2 and two cases N3 patients in distal cholangiocarcinoma patients.)

ExCLNI was identified in 17 of 79 patients (22%) who had nodal involvement. ExCLNI was identified in 14 cases of distal cholangiocarcinoma (36.8%) and 3 cases of hilar cholangiocarcinoma (9.8%). The median number of lymph nodes with ExCLNI was 1 (range 1 to 4).

Correlations between the clinicopathological features and ExCLNI are presented in Table 2. The presence of ExCLNI correlated significantly with the distal bile duct (*p* = 0.002), although there was no significant difference in the number of metastatic lymph nodes in patients with hilar versus distal cholangiocarcinoma (*p* = 0.746).

**Table 2:** Clinicopathological features of significance ExCLNI

		**Patients with lymph nodal metastasis (N = 79)**		
		**Positive ExCLNI N = 17**	**Negative ExCLNI N = 62**	***P Vale***
Age(years)	≦70	11	33	0.426
	>70	6	29	
Gender	Male	13	40	0.4
	Female	4	22	
pT Factor	T1-2	2	23	0.405
	T3-4	15	39	
Perineural invasion	Present	17	55	0.336
	Absent	0	7	
Vascular invasion	Present	6	11	0.102
	Absent	11	55	
Tumor location	Hilar	3	38	0.002
	Distal	14	24	
Histological grade	G1	2	8	0.238
	G2	13	41	
	G3	2	10	
	Others	0	2	
The number of metastatic LN	3≧	9	18	0.086

Seven clinicopathological characteristics of patients with and without ExCLNI are evaluated. ExCLNI: Extra capsular lymph node involvement. LN: lymph node.

### Survival and prognostic impact

There was no significant difference in the survival of patients with hilar versus distal cholangiocarcinoma (*p* = 0.105). Three- and 5-year overall survival rates were 53% and 40%, respectively (median follow-up period: 32 month). Nine independent clinico-pathological variables were analyzed as possible prognostic factors influencing survival in patients with ExHBDCs. On univariate analysis, perineural invasion, vascular invasion, histological grade, and LNM were statistically significant factors (Table 3). Microscopic margin status did not show statistically significant differences on survival. On multivariate analysis, only LNM was identified as a significant independent prognostic factor in patients with resectable ExHBDCs (Table 3).

**Table 3:** Univariate and multivariate analysis for survival

	**Univariate analysis**			**Multivariate analysis**	
	**Overall survival rate (%)**			**Relative risk (95% confidence interval)**	***P value***
**Prognostic factor**	**3 year**	**5 year**	***P value***		
Age					
>70	57	37	*0.376*		
≦70	49	43			
Gender					
Male	52	38	*0.359*		
Female	56	45			
Tumor location					
Hilar	50	32	*0.105*		
Distal	57	51			
pT factor					
pT1/2	66	48	*0.002*	1.15 (0.84-1.57)	*0.387*
pT3/4	45	34			
Lymph node metastasis					
Present	26	14	*<0.001*	1.82 (1.33-2.47)	*<0.0001*
Absent	68	53			
Vascular invasion					
Present	30	22	*<0.001*	1.16 (0.80-1.68)	*0.445*
Absent	59	44			
Perineural invasion					
Present	48	33	*0.009*	1.18 (0.82-1.70)	*0.378*
Absent	76	64			
Histological type					
G1	78	63	*<0.001*	1.27 (0.92-1.76)	*0.146*
G2/G3/others	43	30			
Microscopic margin status					
Positive	57	-	*0.714*		
Negative	53	41			

Predictive prognostic factors influencing survival: nine independent clinicopathological variables were analyzed as possible prognostic factors in patients with ExHBDCs. On univariate analysis, perineural invasion, vascular invasion, histological grade, and lymph node metastasis were statistically significant factors. A multivariate analysis was also performed using the above four factors.

ExHBDCs: extra-hepatic bile duct cancer; pT: 7th classification of AJCC pathological T factor.

**Figure 2:**
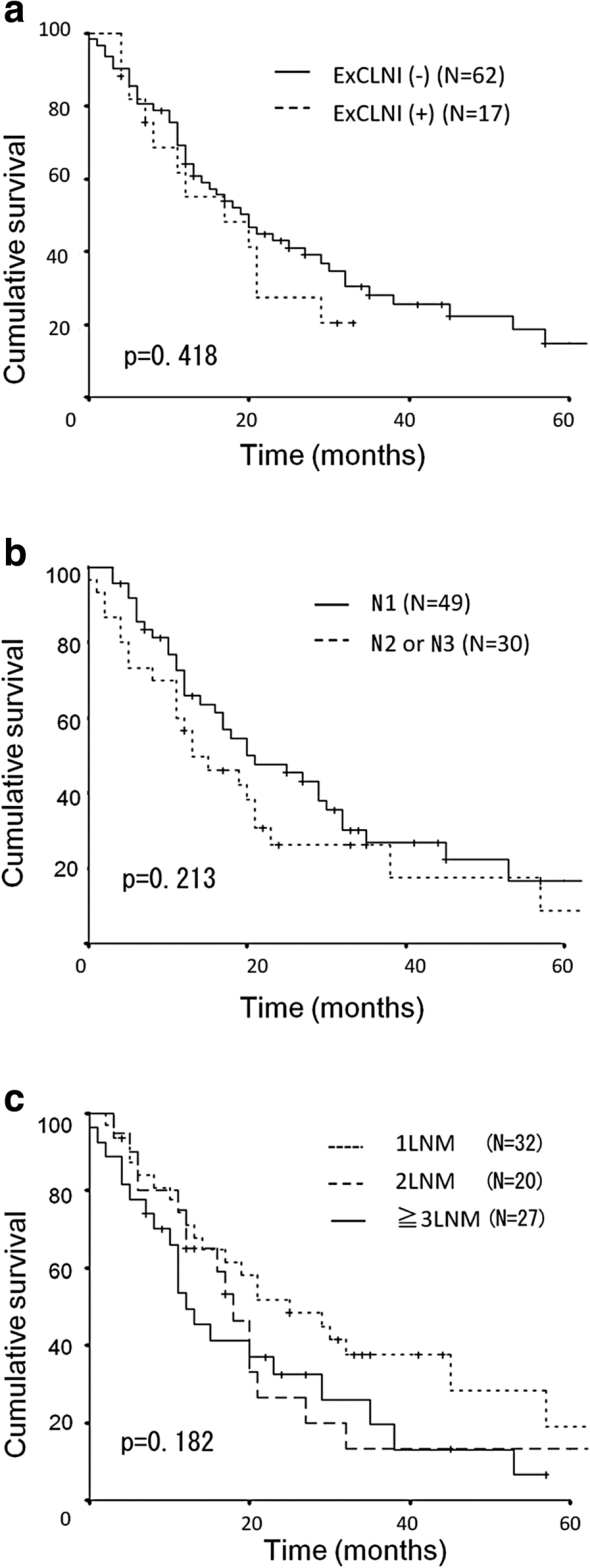
**a, Kaplan-Meier (KM) survival curve of patients with lymph node metastasis (LNM). Patients were divided into two groups: those with and without extra-capsular lymph node involvement (ExCLNI).** There was no significant difference between the two groups (*p* = 0.418). **b**, Kaplan-Meier (KM) survival curve of patients with LNM. Patients were divided into two groups according to location of the LNM (n1, n2, or n3). There was no significant difference between the groups (*p* = 0.213). LNM: Lymph node metastasis **c**, Kaplan-Meier (KM) survival curve of patients with and without ExCLNI. Patients were divided into three groups with ≥3 LNM, 2 LNM, or 1 LNM. There was no significant difference between these groups (*p* = 0.182). ExCLNI: Extra-capsular lymph node involvement; LNM: Lymph node metastasis.

Next, we analyzed whether the subgroups of LNM affect survival. The subgroups of LMN including the presence of ExCLNI, location of the LNM, and the number of LNM had no statistically significant impact on survival (Figure 2a, Figure 2b, and Figure 2c). The subgroups were also analyzed separately in patients with hilar cholangiocarcinoma and patients with distal cholangiocarcinoma with remarkably similar results (data not shown).

## Discussion

In this study, we showed that ExCLNI was present in only 22% of the LNM (7% of overall patients) in patients with surgical treated ExHBDCs. We also revealed that ExCLNI would have no impact on the survival of patients with surgically-treated ExHBDCs.

Our data showed that ExCLNI appears less frequently in patients with resectable ExHBDCs than in patients with adenocarcinoma of the ampulla of Vater or pancreatic cancer (59% and 60%, respectively) [21,22]. One possible factor may be the anatomic features of ExHBDCs, especially in hilar cholangiocarcinoma. Our data showed that incidence of ExCLNI in hilar cholangiocarcinoma was significantly lower than the distal cholangiocarcinoma. Since hilar cholangiocarcinoma surrounds various complex structures such as the portal vein, hepatic artery, and the bile duct, more advanced patients would not have been done operation. Therefore, incidence of ExCLNI in resectable hilar cholangiocarcinoma would be very different from that of resectable distal cholangiocarcinoma. Imre et al. suggested that tumor location significantly associated with ExCLNI in patients with laryngeal and hypopharyngeal cancers [31]. Our study seems to support Imre’s findings. But there seems to be another possible factor, because our data showed that incidence of ExCLNI in distal cholangiocarcinoma was significantly lower than that of previous reported series of Vater carcinoma (32% vs 59%: *p* = 0.030). Certain reason on this difference is still unclear. One possible reason will be differences of biological feature between distal cholangiocarcinoma and Vater carcinoma. The other possible explanation could be that there was also no uniformity in defining ExCLNI which might make it difficult to compare different studies. Wind et al. found at least three definitions of the ExCLNI in the literature [23].

Several authors suggested that ExCLNI reflects cancer progression, and thus, occurs at an advanced tumor stage [32-34]. However, our study did not demonstrate a significant correlation between the occurrences of ExCLNI and advanced p T stages. Van der Gaag et al. showed similar results in Vater carcinoma [21]. They showed that the number of LNM associated with existence of ExCLNI. Our data showed similar result, even though our data did not show statistically significant (*p* = 0.086). The presence of ExCLNI would reflect not cancer progression on primary lesion of tumor (size or depth), but frequency of LNM.

Our data showed that perineural invasion, histological grade, vascular invasion, and LNM were statistically significant prognostic factors in the univariate analysis. Multivariate analysis showed LNM was an independent prognostic factor for survival among these prognostic factors.

A recent report of ExHBDCs revealed that total lymph node count was correlated with disease specific survival [6].They revealed adequate total lymph node count was ≥ 11. In our study, all patients were performed radical lymphadenectomy: skeletonization of the portal vein and hepatic artery and with nodal clearance around the head of pancreas, routinely. Median total lymph nodes counts resected were 14 in our presented study. Therefore we thought that our study had enough qualification to mention about subgroup of LNM in ExHBDCs.

Several authors have adopted the number, location, and LNR as subgroups of LNM [6,9,11,14-16], and they showed these subgroups of LNM had prognostic impact for survival. We thought ExCLNI was also a kind of the subgroup of LNM, and the present study was the first report of analyzing the ExCLNI in patients with ExHBDCs. Our data showed that ExCLNI would not have prognostic impact for survival.

The differences between many previous studies in gastrointestinal cancers showed that ExCLNI was associated with poor prognosis and our data in ExHBDCs may be as follows: not only ExCLNI, but also the other subgroups such as the number, location of LNM would have no impact on the survival of patients with ExHBDCs, because LNM itself would be the most important prognostic factor in ExHBDCs regardless of any subgroup of LNM.

In hilar cholangiocarcinoma, there is a report suggesting that no correlation exists between the degree of lymph node metastasis and prognosis, and that the important factor in determining the prognosis is the presence or absence of lymph node metastasis [2].

A recent report on distal bile duct cancer showed LNM as an independent prognostic factor but that the number of LNM had no impact on survival [7]. Japanese large series of study in distal cholangiocarcinoma also could not find the significant difference between N1 and 2 (location of LNM) [9].

Two reports regarding both bladder carcinoma and oral cancer also suggested that ExCLNI had no prognostic impact [35,36]. In the study evaluating bladder carcinoma, contradictory results occurred because a certain percentage of patients received adjuvant chemotherapy and had a number of lymph nodes removed. In the study evaluating oral cancer, Greenberg et al. suggested that the number of LNMs with ExCLNI had an effect on survival that was unrelated to ExCLNI in and of itself [36].

## Conclusion

ExCLNI was detected in 22% of our patients with LNM in resectable ExHBDCs.

The existence of ExCLNI correlated significantly with the distal bile duct. The presence of ExCLNI and other subgroup of LNM would have no impact on their survival.

## Abbreviations

ExHBDCs: Extra-hepatic bile duct cancers; ExCLNI: Extra capsular lymph node involvement; LNM: Lymph node metastasis; LNR: (metastatic lymph node ratio); TNM: Tumors were classified by the tumor-node-metastasis classification criteria; AJCC: The American Joint Committee on Cancer.

## Competing interests

Authors had no financial competing interests on this article.

## Authors' contributions

TN and MM carried out data collection and analysis, pathological examination, statistical part, and discussion part. KCK, TS and YM carried out pathological diagnosis as pathologist. YA, NK and SH carried out surgery. All authors read and approved the final manuscript.

## References

[B1] Kondo S Hirano S Ambo Y Tanaka E Okushiba S Morikawa T Katoh H Forty consecutive resections of hilar cholangiocarcinoma with no postoperative mortality and no positive ductal margins: results of a prospective study Ann Surg 2004 240 95 101 10.1097/01.sla.0000129491.43855.6b 15213624 PMC1356380

[B2] Nimura Y Kamiya J Kondo S Nagino M Uesaka K Oda K Sano T Yamamoto H Hayakawa N Aggressive preoperative management and extended surgery for hilar cholangiocarcinoma: Nagoya experience J Hepato-Biliary-Pan 2000 7 155 162 10.1007/s005340050170 10982608

[B3] Neuhaus P Jonas S Bechstein WO Lohmann R Radke C Kling N Wex C Lobeck H Hintze R Extended resections for hilar cholangiocarcinoma Ann Surg 1999 230 808 818 discussion 819 10.1097/00000658-199912000-00010 10615936 PMC1420945

[B4] Hidalgo E Asthana S Nishio H Wyatt J Toogood GJ Prasad KR Lodge JP Surgery for hilar cholangiocarcinoma: the Leeds experience Eur J Surg Oncol 2008 34 787 794 10.1016/j.ejso.2007.10.005 18036765

[B5] Kosuge T Yamamoto J Shimada K Yamasaki S Makuuchi M Improved surgical results for hilar cholangiocarcinoma with procedures including major hepatic resection Ann Surg 1999 230 663 671 10.1097/00000658-199911000-00008 10561090 PMC1420920

[B6] Ito K Ito H Allen PJ Gonen M Klimstra D D’Angelica MI Fong Y DeMatteo RP Brennan MF Blumgart LH Jarnagin WR Adequate lymph node assessment for extrahepatic bile duct adenocarcinoma Ann Surg 2010 251 675 681 10.1097/SLA.0b013e3181d3d2b2 20224368

[B7] Choi SB Park SW Kim KS Choi JS Lee WJ The survival outcome and prognostic factors for middle and distal bile duct cancer following surgical resection J Surg Oncol 2009 99 335 342 10.1002/jso.21238 19226533

[B8] Kawasaki S Imamura H Kobayashi A Noike T Miwa S Miyagawa S Results of surgical resection for patients with hilar bile duct cancer: application of extended hepatectomy after biliary drainage and hemihepatic portal vein embolization Ann Surg 2003 238 84 92 12832969 10.1097/01.SLA.0000074984.83031.02 PMC1422661

[B9] Miyakawa S Ishihara S Horiguchi A Takada T Miyazaki M Nagakawa T Biliary tract cancer treatment: 5,584 results from the Biliary Tract Cancer Statistics Registry from 1998 to 2004 in Japan J Hepato-Biliary-Pan 2009 16 1 7 10.1007/s00534-008-0015-0 19110652

[B10] Miyazaki M Kimura F Shimizu H Yoshidome H Otuka M Kato A Yoshitomi H Furukawa K Takeuchi D Takayashiki T One hundred seven consecutive surgical resections for hilar cholangiocarcinoma of Bismuth types II, III, IV between 2001 and 2008 J Hepatobiliary Pancreat Surg 2009 10.1007/s00534-009-0207-2 19936600

[B11] Seyama Y Kubota K Sano K Noie T Takayama T Kosuge T Makuuchi M Long-term outcome of extended hemihepatectomy for hilar bile duct cancer with no mortality and high survival rate Ann Surg 2003 238 73 83 12832968 10.1097/01.SLA.0000074960.55004.72 PMC1422671

[B12] Yonemori A Kondo S Matsuno Y Ito T Nakanishi Y Miyamoto M Tanaka E Hirano S Prognostic impact of regional lymph node micrometastasis in patients with node-negative biliary cancer Ann Surg 2010 252 99 106 10.1097/SLA.0b013e3181e33c0a 20505505

[B13] Yonemori A Kondo S Matsuno Y Ito T Tanaka E Hirano S Prognostic impact of para-aortic lymph node micrometastasis in patients with regional node-positive biliary cancer Br J Surg 2009 96 509 516 10.1002/bjs.6585 19358177

[B14] Yoshida T Matsumoto T Sasaki A Morii Y Aramaki M Kitano S Prognostic factors after pancreatoduodenectomy with extended lymphadenectomy for distal bile duct cancer Arch Surg 2002 137 69 73 10.1001/archsurg.137.1.69 11772220

[B15] Murakami Y Uemura K Hayashidani Y Sudo T Ohge H Sueda T Pancreatoduodenectomy for distal cholangiocarcinoma: prognostic impact of lymph node metastasis World J Surg 2007 31 337 342 discussion 343–334 10.1007/s00268-006-0224-0 17006609

[B16] Kawai M Tani M Kobayashi Y Tsuji T Tabuse K Horiuchi T Oka M Yamaguchi K Sakata Y Shimomura T Yamaue H The ratio between metastatic and examined lymph nodes is an independent prognostic factor for patients with resectable middle and distal bile duct carcinoma Am J Surg 2010 199 447 452 10.1016/j.amjsurg.2009.01.019 19596119

[B17] Alakus H Holscher AH Grass G Hartmann E Schulte C Drebber U Baldus SE Bollschweiler E Metzger R Monig SP Extracapsular lymph node spread: a new prognostic factor in gastric cancer Cancer 2010 116 309 315 10.1002/cncr.24764 19950124

[B18] Metzger R Drebber U Baldus SE Monig SP Holscher AH Bollschweiler E Extracapsular lymph node involvement differs between squamous cell and adenocarcinoma of the esophagus Ann Surg Oncol 2009 16 447 453 10.1245/s10434-008-0248-9 19037700

[B19] Okamoto T Tsuburaya A Kameda Y Yoshikawa T Cho H Tsuchida K Hasegawa S Noguchi Y Prognostic value of extracapsular invasion and fibrotic focus in single lymph node metastasis of gastric cancer Gastric Cancer 2008 11 160 167 10.1007/s10120-008-0473-8 18825310

[B20] Wind J ten Kate FJ Kiewiet JJ Lagarde SM Slors JF van Lanschot JJ Bemelman WA The prognostic significance of extracapsular lymph node involvement in node positive patients with colonic cancer Eur J Surg Oncol 2008 34 390 396 10.1016/j.ejso.2007.05.011 17614246

[B21] van der Gaag NA ten Kate FJ Lagarde SM Busch OR van Gulik TM Gouma DJ Prognostic significance of extracapsular lymph node involvement in patients with adenocarcinoma of the ampulla of Vater Br J Surg 2008 95 735 743 10.1002/bjs.6076 18300268

[B22] Sergeant G Ectors N Fieuws S Aerts R Topal B Prognostic relevance of extracapsular lymph node involvement in pancreatic ductal adenocarcinoma Ann Surg Oncol 2009 16 3070 3079 10.1245/s10434-009-0627-x 19649555

[B23] Wind J Lagarde SM Ten Kate FJ Ubbink DT Bemelman WA van Lanschot JJ A systematic review on the significance of extracapsular lymph node involvement in gastrointestinal malignancies Eur J Surg Oncol 2007 33 401 408 10.1016/j.ejso.2006.11.001 17175130

[B24] Stitzenberg KB Meyer AA Stern SL Cance WG Calvo BF Klauber-DeMore N Kim HJ Sansbury L Ollila DW Extracapsular extension of the sentinel lymph node metastasis: a predictor of nonsentinel node tumor burden Ann Surg 2003 237 607 612 discussion 612–603 12724626 10.1097/01.SLA.0000064361.12265.9A PMC1514520

[B25] Rice TW Blackstone EH Rusch VW 7th edition of the AJCC Cancer Staging Manual: esophagus and esophagogastric junction Ann Surg Oncol 2010 17 1721 1724 10.1245/s10434-010-1024-1 20369299

[B26] Surgery JSoB The general rules for surgical and pathological studies on cancer of the biliary tract, 2nd edition 2004 2 Tokyo: Kanehara

[B27] Noji T Kondo S Hirano S Tanaka E Suzuki O Shichinohe T Computed tomography evaluation of regional lymph node metastases in patients with biliary cancer Br J Surg 2008 95 92 96 17853509 10.1002/bjs.5920

[B28] Noji T Kondo S Hirano S Tanaka E Ambo Y Kawarada Y Morikawa T CT evaluation of paraaortic lymph node metastasis in patients with biliary cancer J Gastroenterol 2005 40 739 743 10.1007/s00535-005-1618-8 16082591

[B29] Hirano S Kondo S Tanaka E Shichinohe T Tsuchikawa T Kato K Matsumoto J Kawasaki R Outcome of surgical treatment of hilar cholangiocarcinoma: a special reference to postoperative morbidity and mortality J Hepato-Biliary-Pan 2009 17 455 462 10.1007/s00534-009-0208-1 19820891

[B30] Nakanishi Y Kondo S Zen Y Yonemori A Kubota K Kawakami H Tanaka E Hirano S Itoh T Nakanuma Y Impact of residual in situ carcinoma on postoperative survival in 125 patients with extrahepatic bile duct carcinoma J Hepatobiliary Pancreat Sci 2010 17 166 173 10.1007/s00534-009-0127-1 19521656

[B31] Imre K Pinar E Oncel S Calli C Tatar B Predictors of extracapsular spread in lymph node metastasis Eur Arch Oto-Rhino-L 2008 265 337 339 10.1007/s00405-007-0464-0 17899142

[B32] Nakamura K Ozaki N Yamada T Hata T Sugimoto S Hikino H Kanazawa A Tokuka A Nagaoka S Evaluation of prognostic significance in extracapsular spread of lymph node metastasis in patients with gastric cancer Surgery 2005 137 511 517 10.1016/j.surg.2005.01.007 15855922

[B33] Di Giorgio A Botti C Sammartino P Mingazzini P Flammia M Stipa V Extracapsular lymphnode metastases in the staging and prognosis of gastric cancer Int Surg 1991 76 218 221 1778719

[B34] Lagarde SM ten Kate FJ de Boer DJ Busch OR Obertop H van Lanschot JJ Extracapsular lymph node involvement in node-positive patients with adenocarcinoma of the distal esophagus or gastroesophageal junction Am J Surg Pathol 2006 30 171 176 10.1097/01.pas.0000189182.92815.12 16434890

[B35] Fleischmann A Thalmann GN Markwalder R Studer UE Prognostic implications of extracapsular extension of pelvic lymph node metastases in urothelial carcinoma of the bladder Am J Surg Pathol 2005 29 89 95 10.1097/01.pas.0000147396.08853.26 15613859

[B36] Greenberg JS Fowler R Gomez J Mo V Roberts D El Naggar AK Myers JN Extent of extracapsular spread: a critical prognosticator in oral tongue cancer Cancer 2003 97 1464 1470 10.1002/cncr.11202 12627511

